# “Reconstruction of segmental defect of flexor tendons of the wrist and hand using extensor digitorum longus”

**DOI:** 10.1080/23320885.2026.2637347

**Published:** 2026-03-10

**Authors:** Koushikk S. Ayyappan, Justin C. R. Wormald, Sarah Tucker, Nakul Kain, Claire Sethu

**Affiliations:** ^a^ University of Oxford, John Radcliffe Hospital, Oxford, UK; ^b^ Nuffield Department of Orthopaedics, Rheumatology and Musculoskeletal Sciences (NDORMS), University of Oxford, Oxford, UK; ^c^ Plastic Surgery Department, John Radcliffe Hospital, Oxford, UK

**Keywords:** Tendons, tendon injuries, tendon transfer, hand injuries

## Abstract

Plastic and hand surgeons rely on traditional tendon grafts, but severe bilateral multi-level trauma may render these unusable. We report a 30-year-old man with extensive forearm loss reconstructed using the entire extensor digitorum longus tendon, nerve and vein grafts, and an ALT flap, demonstrating EDL’s versatility and minimal donor morbidity.

## Introduction

Reconstruction of flexor tendons typically involves tendon autografts. The most common graft options are palmaris longus, plantaris, flexor digitorum superficialis and flexor digitorum longus [1].

However, there are certain instances where the repertoire of tendons can be limited, due to injury, a lack of length, or anatomical variation. In such cases, the extensor digitorum longus (EDL) tendon can be a useful tool and may even confer benefit over the alternatives as a tendon complex that has a similar configuration to that of the extensor digitorum communis (EDC) tendon complex. Here, we report a case of flexor tendon injury where the full EDL tendon was harvested for reconstruction.

## Methods

No ethical approval was required for this study. Written informed consent for patient participation in this case report has been obtained.

## Results

**Figure 1.**
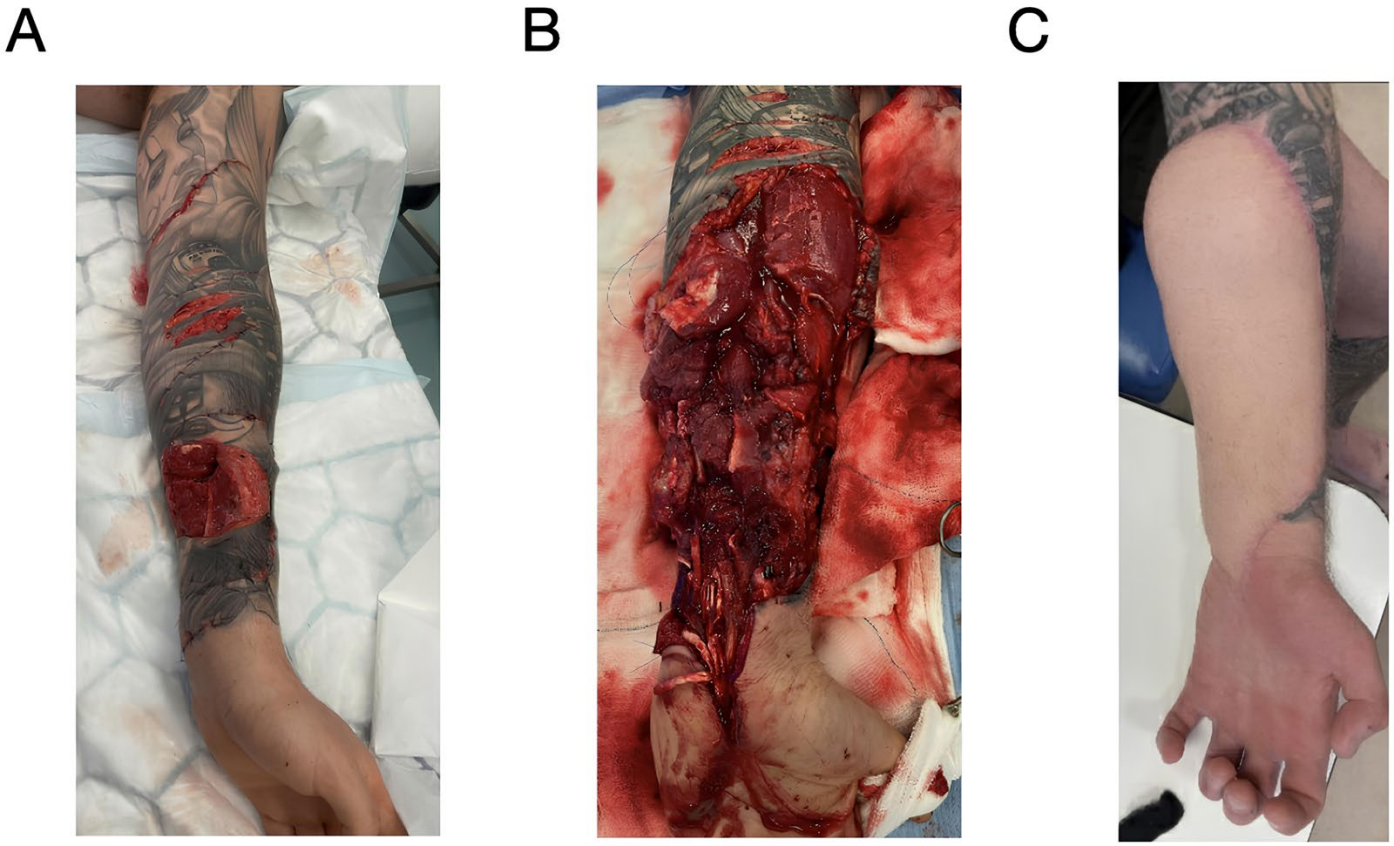
Segmental lacerations in the left arm and wrist. a) Presenting injuries. b) Tendons, nerves and vessels exposed after wound exploration. c) Left forearm post-reconstruction.

A 30-year-old gentleman with a one-year history of depression presented with deliberate self-harm lacerations to the volar aspect of his left forearm after a cannabis-induced psychotic episode. The lacerations extended from just proximal to the wrist crease to the upper arm, and there was a 6 × 3cm skin loss mid-forearm (Figure 1a). Additionally, there were three volar lacerations on the right forearm without significant structural injury. Of note, there were partial injuries to the palmaris longus (PL) on this side.

Following initial debridement of the left forearm, he was noted to have segmental loss of his nerves, arteries, volar superficial veins, and long flexors of the wrist and hand (zone 5 flexor tendon injury) (Figure 1b and Table 1). CT angiography showed a full thickness laceration through the radial artery, and loss of opacification spanning approximately 12 cm. The ulnar artery had a 9 cm loss of opacification. Despite this, there was distal reconstitution of both radial and ulnar arteries, so the hand was well-vascularised.

**Table 1.** Intraoperative findings of nerves, arteries, and tendons.

Structure	Findings
**Nerves**	
Median nerve	14cm loss; present at the proximal third of the forearm, then continues from 3 cm proximal to the carpal tunnel
Ulnar nerve	8cm loss; present to the midpoint of the forearm, then continues from 2 cm proximal to the carpal tunnel
Radial nerve	Not explored. But the proximal segment was identified near its bifurcation
**Arteries**	
Ulnar artery	9cm loss
Radial artery	12cm loss
**Muscles and Tendons**	
Flexor Carpi Radialis (FCR)	12cm loss; proximal third of muscle belly preserved, continues from 5 cm proximal to the carpal tunnel
Flexor Digitorum Profundus (FDP)	8cm loss; proximal half preserved, all tendons preserved distally from 3.5 cm proximal to the carpal tunnel
Flexor Digitorum Superficialis (FDS)	12cm loss; less than half preserved, continues from 2.5 cm proximal to the carpal tunnel
Flexor Carpi Ulnaris (FCU)	3cm loss of tendon; proximal two thirds muscle belly preserved
Flexor Pollicis Longus (FPL)	3cm loss; proximal two thirds preserved
Extensor Carpi Radialis Longus (ECRL)	20% of the tendon cross-section divided and trimmed
Pronator Teres (PT)	Proximal third preserved, but no tendon
Palmaris Longus (PL)	Present but small. Tendon overlies FCU (anatomical variant)

The initial plan for limb reconstruction included: 1) a free anterolateral thigh flap, anastomosed to the radial artery (end-to-end), 2) a short saphenous vein graft for the ulnar artery, bilateral sural nerve cable grafts for the ulnar and median nerves, and 3) a plantaris graft for flexor digitorum profundus (FDP), flexor pollicis longus (FPL) and flexor carpi ulnaris (FCU). However, pre-operative ultrasound demonstrated bilaterally absent plantaris tendons. The contralateral PL was too injured to provide a robust and reliable tendon graft. As such, the left EDL tendon was harvested, for the length of graft that could be obtained.

**Figure 2.**
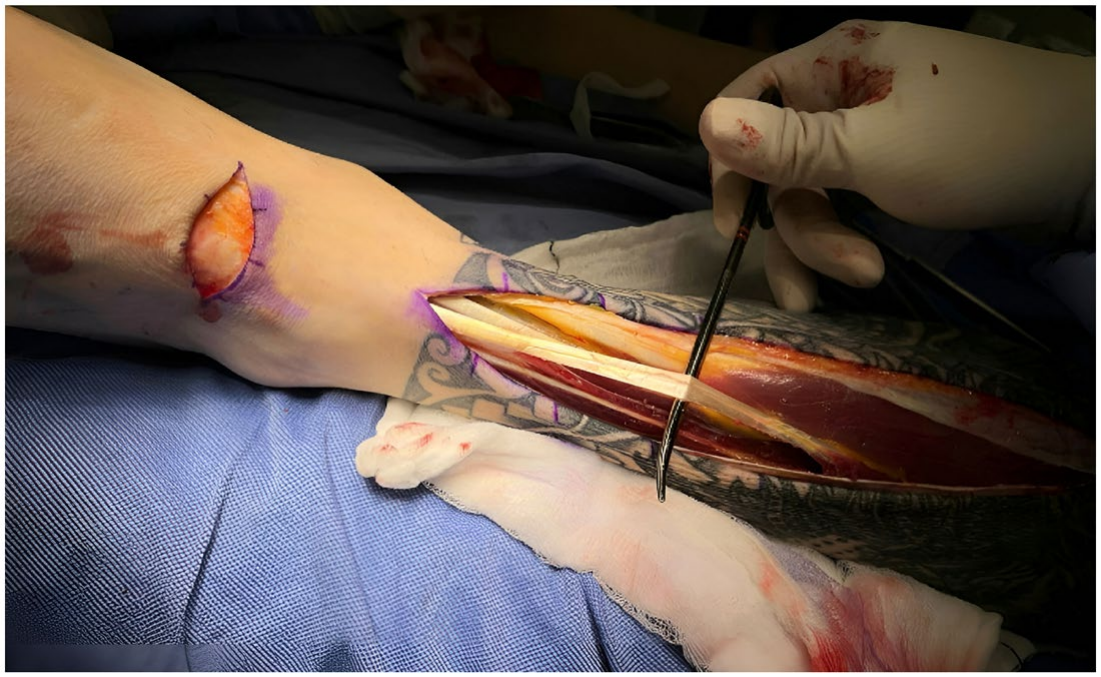
Dissection of the distal lower leg showing the Extensor Digitorum Longus (EDL) separated from adjacent muscles and tendons in the anterior compartment (distal on the left, proximal on the right).

For the EDL harvest, the dorsal metatarsophalangeal joints and distal retinaculum were used as anatomical landmarks to make longitudinal incisions on the anterior leg. The EDL was identified to be distinct from the extensor digitorum brevis (EDB) distally by tracing the tendon to the dorsal digital expansion at the metatarsophalangeal joints, where the EDB converges with the EDL. The EDL was then separated (Figure 2), divided with care, and harvested up to its muscle belly.

The flexor tendons of the fingers (FDP and FDS) were reconstructed using the four tendons of the EDL graft with a side-to-side Bunnell-style tendon repair, chosen for its superior biomechanical strength and comparable bulk to a Pulvertaft weave [2,3]. This approach reduces friction and adhesion risk and allows coordinated finger flexion through a shared proximal tensioning point at the FDP musculotendinous junction. The first EDL tendon was secured to the FDP and FDS to the index finger, the second secured to the FDP and FDS to the middle finger, third to the FDP to the fourth and fifth fingers, and fourth to the FDS to the fourth and fifth fingers. Despite the increased tendon bulk and potential for higher friction, impaired gliding and adhesion formation [4], the EDL was sutured to both the FDS and FDP for the index and middle fingers because these digits contribute disproportionately to pinch, power and fine motor control. FDS restoration improves force tolerance [5], range of motion [6], and allows for independent proximal interphalangeal joint flexion, providing a smooth gliding surface for FDP [7]. Tension was set proximally by securing to the FDP musculotendinous junction, which ensured movement of all four distal grafts. The distal stumps of FDP and FDS were sutured together to enhance flexor function. The remnant redundant proximal part of the EDL tendon was used to reconnect the FPL, FCR and FCU muscle bellies to their respective distal tendons.

In the postoperative phase, the patient generally recovered well with no complications (Figure 1c shows the forearm post-reconstruction). The patient was seen every 3 days by physiotherapy until discharge. He was seen by hand therapy every 5–10 days until 2 weeks post-op, then every 3–4 weeks.

Two days post-op, the patient was sitting independently but needed a hoist and two-person assistance for transfers and mobilisation. Three days post-op, the patient showed a left foot drop and struggled to weight balance. Ankle flexion was weak and with pain; knee extension was achieved, but flexion was only 20°. Exercises included regular ankle pumps to increase the range of motion to try reach plantigrade. The initial goal was for the patient to achieve step transfers with a gutter frame and two-person assistance by 1 week post-op, progressing to independent transfers by 10 days post-op.

At five days post-op, active ankle dorsiflexion was 50% of the normal range, with 4/5 MRC power. A plantigrade position whilst sitting was achieved, and knee flexion improved to 30°. In addition, hand therapy began with passive movements. Finger extension was achieved through active-assisted range of motion, while flexion was passive and limited to a near full hook. A below-elbow split soft cast was applied, with a dorsal blocking hood to prevent excessive finger extension, and a window to monitor the flap. This anti-claw splint held the metacarpophalangeal (MCP) joints at 60° in increased flexion, and interphalangeal joints (IPJs) in extension. A cuff splint was also made for intrinsic muscle exercises. The patient was advised to practice pen grip tasks and active hook exercises for distal interphalangeal joint (DIPJ) activation.

At one week post-op, both 90° knee flexion and 75° active foot dorsiflexion were achieved. The patient’s goal was now to independently manage a full flight of stairs with a gutter frame or elbow crutch by post-op day 10. Hand assessment showed a reduction in power in the distal and middle third of the forearm, with a weak grip and 2/5 power for finger and thumb flexion and extension. It was predicted that the patient would return to the gym in 3 months’ time with physiotherapy. However, it was predicted he would never return to boxing, and we explained this to the patient.

At 10 days post-op, using an elbow crutch, the patient could mobilise 30 m unaided and ascend the stairs with a single handrail. Leg extensor function was almost fully restored, and the foot drop was minimal. The goal now was to wean from the elbow crutch to return to work, hobbies and the gym. Hand assessment showed the patient was able to maintain finger position after passive placement. The range of motion for passive flexion (proximal interphalangeal joints; PIPJ 92–98°, DIPJ 55–70°) was greater than active flexion (MCPJ 62–68°, PIPJ 60–79°, DIPJ 10–35°). Thumb flexors were strong (FPL 5/5), but posture and opposition were limited. The patient was discharged that day and given a volar hand night splint with Velcro straps to hold the digits in place. During the day, the patient would wear the anti-claw splint as per usual.

The patient showed excellent progression and recovery. At 1 month post-op, the patient could perform light grasp activities, such as picking up cones and household objects using lateral pinch. Full passive global finger flexion was possible. Additionally, the patient reported constant tingling in digits and reduced sensation, which was managed with gabapentin. At 2 months-post-op, the patient had returned to the gym for push activities. He could hold a 3 kg shopping bag with an isometric hook grip for a minute without fatigue, but the intrinsic muscles were still weak. Exercises included resting the wrist in extension and targeting finger extension and hook motions. Gabapentin was stopped, and recovery in sensation was tracked clinically with progressive Tinel’s signs in both the median and ulnar nerve distributions. At this stage, there was a + Tinel’s sign for the ulnar nerve 5 cm proximal to the wrist crease, and 6 cm proximal to the carpal tunnel region for the median nerve.

At 3 months post-op, the patient returned fully to work and the gym, and there were no restrictions placed. However, he struggled with composite flexion and fine grip e.g. holding cables. FDP power improved to 3/5 for all fingers, whereas FDS remained at 2/5 and lumbricals 1/5. Exercises now include thumb IPJ flexion and extension, interlocking fingers, abducting the fifth digit, and retropulsing the thumb in extension. The goal was to aim for a half-range composite flexion grip (half-fist) in the anti-claw splint, and full finger extension. Sensation was still lacking in the volar hand but returned to the dorsal hand and 4th webspace. This suggested that the median nerve was struggling to regenerate, also supported by the thenar eminence atrophy seen.

At 4 months post-op, the patient could achieve a half-fist. Active finger flexion was still reduced at the PIPJs (68–75°) and DIPJs (20–42°). The patient was unable to place and hold the lumbricals, as the IPJs tended to flex. Exercises focused on strengthening these intrinsic muscles with a cuff splint. Tinel’s was now positive for the median nerve 4 cm proximal to the distal carpal tunnel, and 4 cm proximal to the carpal crease for the ulnar. At 5 months post-op, Tinel’s was positive for the median nerve 8 cm distal to proximal wrist crease, and ulnar nerve 7.5 cm, with symptoms into the hypothenar eminence and fifth digit, suggesting progressive nerve regeneration.

At 7 months post-op, muscle bulk returned on the thenar eminence, and the patient could now drive and box, with good punches and no pain upon contact (Supplementary Video 1). The patient could make a three-quarters fist in the anti-claw splint, and place and hold the lumbricals. He still reported persistent altered sensation with pins and needles, but there was a meaningful sensory recovery throughout the hand. We expect the patient to improve IPJ extension to around 20° for all fingers and eventually make a full fist. There was no evidence of donor site morbidity at the lower leg, with preserved gait, ankle stability and toe movements.

At the most recent follow-up appointment, the patient reported tightness preventing full extension of all joints. We plan to perform a left forearm thinning of the fasciocutaneous flap and flexor tenolysis, for which the patient is currently on the waiting list for.

## Discussion

We present a case of segmental flexor tendon injury in the left arm and hand. The typical upper limb tendon grafts, such as FDS, could not be used due to injury in the left forearm. The contralateral arm was also avoided due to injury, and since tendons such as PL and FDS provide limited graft length (e.g. PL is half the length of EDL) [8]. Additionally, plantaris can be absent in ≈10% of the population [9], as in this patient. Therefore, the EDL tendon was selected as the donor autograft. EDL tendons are approximately 21-32cm in length [8,10] in their entirety, making them well-suited for use in multiple tendon reconstructions, particularly where the tendons to be reconstructed have some interconnection, as EDC does (compare this to FDL, which is around 6.7 cm [11]).

The literature describes several issues with full-length EDL grafts. Firstly, harvest is difficult because the EDL lies deep to the extensor retinaculum and lateral to the extensor hallucis longus and tibialis anterior tendons [12]. The presence of two extensor retinacula and extensive network juncturae between adjacent EDL tendons results in adherence to fascia and subcutaneous tissue. Hence, extensive dissection is required, resulting in potential donor-site morbidity, particularly affecting toe extension. To mitigate this, other groups have developed modified techniques involving partial harvest of EDL entirely proximal to the superior extensor retinaculum [12]. However, this approach yields a limited graft length (18.5 cm vs the full 32 cm) and prevents the isolation of individual EDL tendon slips. As the fifth toe lacks an extensor digitorum brevis slip, it may lose extension if the EDL is weakened [12].

Furthermore, EDL is an extrasynovial graft, which may lead to greater adhesions, peritendinous necrosis, reduced tendon excursion, and limited angular joint range of motion relative to intrasynovial grafts [13–15], potentially related to differences in glycoprotein, DNA and protein synthesis [16]. Nevertheless, there is limited clinical evidence demonstrating the superiority of intrasynovial grafts (samora), perhaps as extrasynovial grafts have improved tensile properties [17]. The use of extrasynovial grafts can lead to issues during rehabilitation, such as difficulty in full flexion or extension of the digits, as our patient experienced. In our case, the limited range of motion likely reflects reduced tendon excursion, as the graft was attached more proximally to the muscle belly than the tendon’s typical insertion. This was due to distal muscle loss and absent tension, requiring a longer graft to restore functional length.

In this case, due to limited options, the full EDL was carefully harvested and grafted, and the patient showed full recovery of power and mobility. This demonstrates that careful surgical technique and physiotherapy can still lead to successful outcomes.

## Conclusions

This case presents a valuable technique to be added to the toolbox of plastic surgeons. Whilst uncommon, in some instances, anatomical variation such as the absence of plantaris, or forearm trauma may limit the tendons typically available for grafting. This report demonstrates that the entire EDL is an excellent option for multiple tendon reconstructions and can be harvested with minimal donor site morbidity - despite the literature’s concerns with the extensor retinacula and juncturae tendinum.

## Supplementary Material

supplementary for review.zip

Appendix supplementary legend.docx

## Data Availability

Not applicable.

## Appendix

**Supplementary Video 1**: Patient in black shirt demonstrates full range of motion and power whilst boxing.
