# Supplementary material for: “Reconstruction of segmental defect of flexor tendons of the wrist and hand using extensor digitorum longus”
Source: Case Reports Plast Surg Hand Surg. 2026 Mar 10;13(1):2637347. doi: 10.1080/23320885.2026.2637347 (PMC12978179; doi:10.1080/23320885.2026.2637347)
Supplement: supplementary for review.zip [file ICRP_A_2637347_SM7093.zip › Appendix supplementary legend.docx]

**Appendix**

Supplementary Video 1: Patient in black shirt demonstrates full range of motion and power whilst boxing.
